# Media use and organ donation willingness: A latent profile analysis from Chinese residents

**DOI:** 10.3389/fpubh.2022.1000158

**Published:** 2022-10-17

**Authors:** Fangmin Gong, Yuhan Jia, Jinzi Zhang, Meiling Cao, Xiaocen Jia, Xinying Sun, Yibo Wu

**Affiliations:** ^1^School of Literature and Journalism Communication, Jishou University, Jishou, China; ^2^School of Humanities and Social Sciences, Harbin Medical University, Harbin, China; ^3^Affiliated Hospital of Integrated Traditional Chinese and Western Medicine, Nanjing University of Chinese Medicine, Nanjing, China; ^4^School of Public Health, Qingdao University, Qingdao, China; ^5^School of Public Health, Peking University, Beijing, China

**Keywords:** media use, organ donation, willingness to accept, cross-sectional study, latent profile analysis

## Abstract

**Background:**

Previous studies have paid attention to media as an important channel for understanding organ donation knowledge and have not divided groups according to the degree of media use to study their differences in organ donation. Therefore, the purpose of this study is to explore the influence of media use on organ donation willingness and the influencing factors of organ donation willingness of people with different media use levels.

**Methods:**

A cross-sectional study of residents from 120 cities in China was conducted by questionnaire survey. Using Mplus 8.3 software, the latent profile analysis of seven media usage related items was made, and multiple linear regression was performed to analyze the influence of varying levels of media use on organ donation willingness of different population.

**Results:**

All the interviewees were divided into three groups, namely, “Occluded media use” (9.7%), “Ordinary media use” (67.1%) and “High-frequency media use” (23.2%). Compared with ordinary media use, high-frequency media population (β = 0.06, *P* < 0.001) were positively correlated with their willingness to accept organ donation, residents who used media occlusion (β = −0.02, *P* < 0.001) were negatively correlated with their willingness to accept organ donation. The influencing factors of residents' accept willingness to organ donation were different among the types of occluded media use, ordinary media use and high-frequency media use.

**Conclusion:**

It is necessary to formulate personalized and targeted dissemination strategies of organ donation health information for different media users.

## Introduction

Organ transplantation is an effective method for the treatment of end-stage organ failure, which is widely practiced around the world (1). The organ donation rate per million population had increased from 2.01 in 2015 to 3.70 in 2020 (2). By July 15, 2022, the number of organ donation volunteers in China has reached more than 4.85 million, the number of organ donations has reached more than 40,000, and the number of donated organs has reached 120,000 (3). Even so, an ongoing challenge for organ donation and transplantation is that the demand for organs far exceeds the supply (4). In most countries, organ donation is carried out based on the prior consent of donors or his or her close relatives upon death (5). Public attitudes toward living organ donation and transplantation are very important, however, their awareness is inadequate or biased, which may hinder the development of organ donation and transplantation, such as in Belgium, Spain, China and Australia (6–9). The media plays a key role in establishing a newsworthy story agenda for the society. It has become the main information source for the public to know about organ donation by establishing the topic and publicity channels of organ donation in various ways (10, 11). The longer people use the media, the more information they get. It increases people's sensitivity to organ donation, strengthens their motivation to donate and promotes practical action. By analyzing the content of organ donation on TV, Brian L. Quick found a positive influence on the actual transplant rate during the period of 1990–2005 (12). Andrew M. Cameron used social networks for organ donor registration, and found that the number of online organ donation registrations in the United States increased by 2,200%. In some states (such as Georgia), the number of registered people increased by 12,000%, and even in the state with the lowest response rate (such as Hawaii), it increased by 800% (13). Greg Moorlock and Heather Draper found that social media can be used to arouse people's sympathy for organ donation and promote organ donation by using identifiable victim effect by exploring three methods of organ donation (14). While promoting organ donation, mass media may bring to a pretty pass due to negative or sensational reports (15, 16). For example, Polish national newspapers, tabloids and TV news programs reported a series of negative events related to transplantation in 2007, followed by the number of transplantation dropped by 56% 2.5 months after the report (17). A similar thing happened in Australia from 1989 to 2003, when the number of donors dropped from 14 to 9 (18, 19).

Although previous studies have paid attention to the relationship between media and organ donation, they have not divided groups according to the degree of media use to study the differences in organ donation. Studies have shown that when the audience gets information, they will form three groups of people. First, news avoiders who do not contact the mass media (20). Second, people who contact information through various mass media (21). Third, people who are exposed to information only through new media or traditional media (22). There is obvious group heterogeneity in the use of media, and the information of organ donation obtained by different groups is also uneven (23). Latent Profile Analysis (LPA) was used to identify information seeking attributes and patterns, and to classify people into different profiles (i.e., types) (24). In this person-centered approach, mass media usages and interpersonal communication patterns are treated as information seeking characteristics of different types of people (25). LPA can identify the media use types of different groups of people, and accurately analyze the related factors that affect the public's willingness to accept organ donation. By identifying the media usage types of different groups of people through LPA, we can accurately analyze the related factors that affect the public's willingness to accept organ donation, so as to achieve accurate communication and enhance the public's willingness to accept organ donation. Therefore, this study adopted the individual-centered latent variable method to identify the media use types of different groups of people through latent profile analysis. The purpose was to explore the relationship between media use patterns and people's willingness toward organ donation, find out the factors that affect the public's willingness and put forward valuable suggestions for improving their donation willingness.

## Methods

### Ethics statement

The study protocol was approved by the Institutional Review Committee of Jinan University (JNUKY-2021-018), Guangdong, China. All respondents have informed consent and voluntarily participated in the survey.

### Data source and sample

Inclusion criteria for this study: (1) The nationality of the People's Republic of China; (2) Age ≥12 years; (3) China's permanent resident population (annual travel time ≤ 1 month); (4) Participate in the study voluntarily and fill in the informed consent form; (5) Participators can complete the network questionnaire survey by themselves or with the help of investigators; (6) Participate can understand the meaning of each item in the questionnaire.

Exclusion criteria include: (1) inconvenient movement, confusion, mental disorders; (2) Those who are participating in other similar research projects; (3) People are unwilling to cooperate.

### Survey method

Multi-stage sampling was used. First, the provincial capitals and four municipalities of 23 provinces and five autonomous regions in China were directly included, and 2–6 cities, a total of 120 cities, were selected from the non-provincial-level administrative regions of each province and autonomous region by random number table method. At least one investigator or one investigation team ( ≤ 10 people) were openly recruited in these cities. Based on the results of the seventh national census, the residents of these 120 cities were sampled with quotas (the attributes of quotas are gender, age, urban and rural distribution), which basically accords with the population characteristics of China. With the help of Questionnaires Platform, the investigators distributed questionnaires to the public one-on-one, and the respondents answered by clicking the link, and the investigators entered the questionnaire number. If the respondent has thinking ability but not enough action ability to answer the questionnaire, the investigator will query and fill in the questionnaire instead of him. The survey was conducted from July 10, 2021 to September 15, 2021.

### Measurement

The questionnaire included social demographic information (such as gender, age, ethnicity and education level), media use, social support, depression, anxiety, pressure and willingness to accept organ donation. Among them, media use, pressure and willingness to accept organ donation were self-designed scales, while social support, depression and anxiety were international general scales.

The research team designed the questionnaire after consulting books and literature scientifically and comprehensively. Before the questionnaire was officially used, experts consulted and discussed on June 7, June 11, June 15, June 18, July 3, and July 8, 2021. The consulted experts were all senior professional titles and regional representatives. Specialties include social medicine, health education, health statistics, health management, psychology, humanities, journalism and communication, pharmacy, nursing, sociology, philosophy, etc.

### Scale of willingness to accept organ donation

Residents' willingness to accept organ donation was reported by the residents themselves (26). Use a score from 0 to 100. The higher the score, the stronger the will power.

### Self-made media usage scale

The self-made media usage scale was used to measure the type and degree of media usage. Through scientific and comprehensive access to books and literature, the research team designed the questionnaire (27, 28), and experts (all with senior titles and regional representation) were consulted and discussed to ensure that the questionnaire is applicable to all media users. There were items items in the scale, which were used to know the contact frequency of respondents to seven kinds of media: newspapers, magazines, radio, television, books (non-textbooks), personal computers (including tablets) and smart phones. Each entry was set with five options: never use, occasionally use, sometimes use, often use and almost every day, which were assigned to 1–5 in turn (never use = 1, almost every day = 5). The number of days that the measured person was exposed to various media in one week was used as the scoring basis, and the total score of each option was added as the scoring result, with a total score of 35 points. A higher score indicates that the subjects' media usage was higher. The Cronbach's alpha of the scale was 0.70.

### Perceived social support scale

The PSSS was used to measure social support (29). PSSS was a 12-item self-report that assessed emotional support from friends, family and significant others. There were seven options in this scale, from “extremely disagree” to “extremely agree”, which were assigned 1–7 in turn (extremely disagree = 1, extremely agree = 7). The respondents scored the degree of consent of each item, and the scores of all items were added together to get a score between 12 and 84, which reflected the total degree of social support felt by the individual. The higher the score, the higher the degree of support. The Cronbach's alpha of the scale was 0.96.

### 9-item patient health questionnaire

The depression was measured by 9-Item Patient Health Questionnaire (30). The subjects' self-assessment based on their past two weeks' situation and the depression assessment based on the self-assessment scores have good reliability and validity in assisting the diagnosis of depression and assessing the severity of symptoms. The scale consists of 9 items. For each item, four options were set: almost nothing, a few days, more than half, and almost every day. The score was assigned to 0–3 (almost nothing = 0). The total score of each option was added as the scoring result, and the total score was 27. The higher the score, the more prone to depression. The Cronbach's alpha of the scale was 0.94.

### 7-item generalized anxiety disorder

The anxiety was measured by 7-item generalized anxiety disorder (GAD-7). The subjects made self-evaluation based on their own situation in the past two weeks, and evaluated anxiety disorder according to the results of self-evaluation scores. GAD-7 had good reliability, as well as criterion, construct, factorial, and procedural validity (31). There are seven items in the scale. For each item, four options were set: none at all, a few days, more than half, and almost every day. The score was assigned to 0–3 (none at all =0). The total score was 21 points. The higher the score, the more anxious you were. The Cronbach's alpha of the scale was 0.96.

### Self-made pressure scale

The self-made pressure scale was used to measure the pressure (32). Self-evaluation of personal pressure by subjects. The scale was scored by six points, and the subjects scored from 1 to 6 according to their perceived level. The higher the score, the more obvious the pressure. The scoring method was mainly the addition of three self-rated scores, which was the level of personal pressure. The measurement mainly focuses on the individual's ability to deal with pressure, taking time as a unit, from 2weeks to 1 year to perceive and evaluate the pressure in life (including family and work). There were three questions in total. The Cronbach's alpha of the scale was 0.86.

### Statistical analysis

Continuous variables were expressed by M±SD, Chi-square (*x*^2^) test was used for comparison between groups, and classified variables were expressed by frequency. The potential profile of seven items used by media was analyzed by Mplus8.3 software, the smaller the values of Akaike Information Criteria (AIC) and Bayesian Information Criteria (BIC) were, the better the LPA fitting model was. The entropy value was between 0 and 1, and the closer to 1, the more accurate the classification. The significant difference between Lomendell-rubin (LMR) and Bootstrap Likelihood Ratio Test (BLRT; *P* < 0.05) indicates that K-type model was superior to K-1 model. Gradually increase the number of categories in the model from the initial model until the model with the best fitting data was found. On the basis of retaining the best category model, SPSS26.0 software was used for stepwise regression analysis. *P* < 0.05 (two-side) is statistically significant.

## Results

### Analysis of potential profile of media use

Selected 1–6 potential profile models to analyze the frequency of media usage. The results showed that the values of AIC, BIC, and aBIC decreased with the increase of the number of classifications. The two indexes of LMR and BLRT (*P* < 0.001) showed that the models of Class 2, Class 3, Class 4 and Class 5 fit well, and the value of Entropy was closest to 1 when it was in Class 4, followed by Class 3. Combined with the model diagrams of various categories, the classification models of three potential categories (C1, C2, and C3) were finally selected as the classification of residents' media usage frequency. The average probability of residents belonging to each category was between 95 and 98%, indicating that the results of the three models are reliable, as shown in Table 1.

**Table 1:** Potential profile model fitting indicators of media usage types.

**Model**	**K**	**AIC**	**BIC**	**aBIC**	**Entropy**	**LMR**	**BLRT**	**Category probability (%)**
1	14	246944.918	247047.237	247002.746				1
2	22	230380.614	230541.400	230471.487	0.919	< 0.001	< 0.001	0.747/0.253
3	30	221958.644	222177.898	222082.562	0.948	< 0.001	< 0.001	0.097/0.672/0.231
4	38	216424.795	216702.517	216581.758	0.959	< 0.001	< 0.001	0.089/0.115/0.668/0.128
5	46	208110.241	208446.430	208300.248	0.943	< 0.001	< 0.001	0.298/0.207/0.262/0.134/0.098
6	54	207582.155	207976.812	207805.207	0.985	0.994	1.000	0.449/0.080/0.080/0.239/0.055/0.098

**Figure 1:**
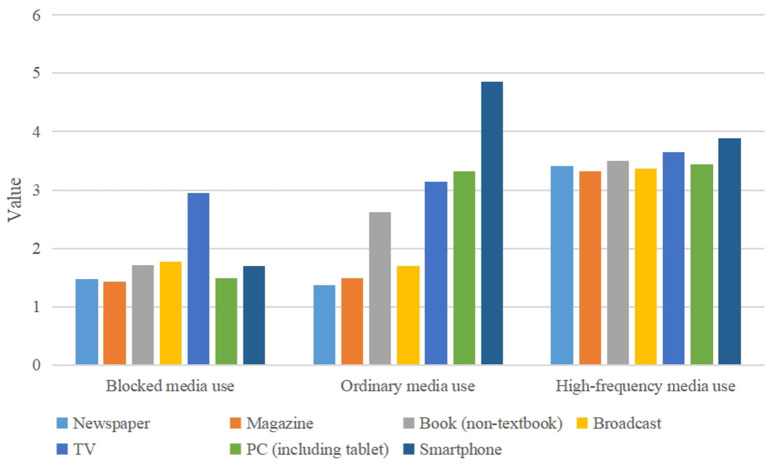
Profile of three potential categories of residents' media use of seven items.

There were obvious differences in the scoring probability of the three potential categories in seven media usage items, showing different characteristics. The most obvious characteristics were judged according to the dimensional differences within and between groups. The subjects in category C2 account for about 67.1% of the total subjects, and the frequency of media use (18.504 ± 2.643) was higher than that in category C1 but lower than that in category C3. Therefore, this category was named “Ordinary media use”. Category C1 subjects accounted for about 9.7% of the total subjects, and the scores of each item (12.515 ± 1.788) were not high, and were significantly lower than those of C2 and C3. According to its scoring characteristics, this category was named “Occluded media use”. Category C3 subjects accounted for about 23.2% of all subjects, and its score (24.571 ± 3.510) was significantly higher than that of C1 and C2. Therefore, this category was named as “High-frequency media use” (see Figure 1).

### Descriptive statistics and one-way ANOVA

A total of 11,031 questionnaires were collected. Among the participants, 5,998 (54.4%) were females, 4,665 (42.3%) were younger than 30 years old, 6,360 (57.7%) were non-agricultural registered permanent residence, 8,008 (72.6%) were urban residents, and 6,487 (58.8%) had college degree or above (see Table 2).

**Table 2:** Descriptive statistics and one-way ANOVA.

**Category**	**All Comers**	**Ordinary media use**	**Occluded media**	**High-frequency media**	***x^2^***	***P***
	**(*N* = 11031, 100%)**	**use (N1 = 1067, 9.7%)**	**use (N2 = 7415, 67.1%)**	**use (N3 = 2549, 23.2%)**	
Gender					117.2	< 0.001
Female	5,998 (54.4)	538 (50.4)	4,268 (57.6)	1,192 (46.8)		
Male	5,033 (45.6)	529 (49.6)	3,147 (42.4)	1,357 (53.2)		
Age					634.5	< 0.001
≤ 18	1,065 (9.7)	109 (53.0)	772 (63.3)	184 (65.3)		
19–40	5,332 (48.3)	257 (55.2)	3,829 (60.6)	1,246 (63.1)		
41–65	3,759 (34.1)	318 (48.2)	2,570 (50.3)	871 (61.1)		
≥66	875 (7.9)	383 (39.7)	244 (42.9)	248 (52.6)		
National minorities	645 (5.8)	66 (6.2)	412 (5.6)	167 (6.5)		
Permanent residence					224.3	< 0.001
Rural	3,023 (27.4)	571 (53.5)	1,857 (75.0)	670 (73.7)		
Urban	8,008 (72.6)	496 (46.5)	5,558 (25.0)	1,879 (26.3)		
Household registration permit					187.6	0.010
Agriculture	4,671 (42.3)	442 (41.4)	3,018 (59.3)	1,028 (59.7)		
Non-agriculture	6,360 (57.7)	625 (58.6)	4,397 (40.7)	1,521 (40.3)		
Education level					559.3	< 0.001
Primary school and below	1,127 (10.2)	453 (42.5)	481 (6.5)	193 (7.5)		
Middle school	3,417 (31.0)	340 (31.9)	2,334 (31.5)	743 (29.2)		
College level or above	6,487 (58.8)	274 (25.7)	4,600 (62.0)	1,613 (63.3)		
Marital status					323.7	< 0.001
Unmarried	4,363 (39.6)	263 (24.7)	3,115 (42.1)	985 (38.7)		
Married	6,226 (56.4)	658 (61.7)	4,089(55.1)	1,479 (58.0)		
Divorced	207 (1.9)	14 (1.3)	142 (1.9)	51 (2.0)		
Widowed	235 (2.1)	132 (12.4)	69 (0.9)	34 (1.3)		
Number of houses owned					376.3	< 0.001
0	1,083 (9.8)	151 (14.2)	618 (8.3)	314 (12.3)		
1	6,598 (59.8)	713 (66.8)	4,493 (60.6)	1,392 (54.6)		
2	2,440 (22.1)	146 (13.7)	1,706 (23.0)	588 (23.1)		
≥3	910 (8.3)	57 (5.3)	598 (8.1)	255 (10.0)		
Family economic status					189.5	< 0.001
≤ 6000	7,500 (68.0)	861 (80.7)	5,061 (68.3)	1,578 (61.9)		
6001–12000	2,769 (25.1)	162 (15.2)	1,886 (25.4)	721 (28.3)		
>12000	762 (6.9)	44 (4.1)	468 (6.3)	250 (9.8)		
Whether have children					432.7	< 0.001
No	5,062 (45.9)	306 (28.7)	3,600 (48.6)	1,156 (45.4)		
Yes	5,969 (54.1)	761 (71.3)	3,815 (51.4)	1,393 (54.6)		
Whether have brothers or					206.4	< 0.001
No	2564 (23.2)	178 (16.7)	1746 (23.5)	640 (25.1)		
Yes	8,467 (76.8)	889 (83.3)	5,669 (76.5)	1,909 (74.9)		
Whether have debts					125.6	0.001
No	6,780 (61.5)	791 (74.1)	4,381 (59.1)	1,608 (63.1)		
Yes	4,251 (38.5)	276 (25.9)	3,034 (40.9)	941 (36.9)		
Whether have medical insurance					115.7	< 0.001
No	2,299 (20.8)	224 (21.0)	1,507 (20.3)	568 (22.3)		
Yes	8,732 (79.2)	843 (79.0)	5,908 (79.7)	1,981 (77.7)		
Whether have religious beliefs					142.5	< 0.001
No	10,709 (97.1)	1,019 (95.9)	7,214 (97.3)	2,476 (97.1)		
Yes	321 (2.9)	48 (4.5)	201 (2.7)	73 (2.9)		
Depression					601.6	< 0.001
No depression	5,031 (45.6)	496 (46.5)	3,671 (49.5)	864 (33.9)		
Mild depression	3,801 (34.5)	384 (36.0)	2,722 (36.7)	695 (27.3)		
Moderate depression	1,148 (10.4)	116 (10.9)	672 (9.1)	360 (14.1)		
Moderate to severe depression	803 (7.3)	56 (5.2)	273 (3.7)	474 (18.6)		
Severe depression	248 (2.2)	15 (1.4)	77 (1.0)	156 (6.1)		
Anxiety					457.3	< 0.001
No anxiety	6,170 (55.9)	571 (53.5)	4,542 (61.3)	1,057 (41.4)		
Mild anxiety	3,364 (30.5)	358 (33.6)	2,324 (31.3)	682 (26.8)		
Moderate anxiety	1,198 (10.9)	94 (8.8)	317 (4.3)	389 (15.3)		
Severe anxiety	299 (2.7)	44 (4.1)	232 (3.1)	421 (16.5)		
Pressure					746.1	< 0.001
Mild pressure	2,719 (24.6)	251 (23.5)	1,946 (26.2)	522 (20.5)		
Moderate pressure	7,653 (69.4)	704 (66)	5,217 (70.4)	1,732 (67.9)		
Severe pressure	659 (6.0)	112 (10.5)	252 (3.4)	295 (11.6)		

Based on the analysis of the potential profile of media use, it was found that the number of residents aged less than 30 (55.4%) was the largest among the ordinary and high-frequency media users. Among the media- occluded people, the majority (42.5%) had primary school education or below. In the cases of no depression (49.5%), no anxiety (61.3%), and mild stress (26.2%), people with ordinary contact with media accounted for more, while in the cases of severe depression (6.1%), severe anxiety (6.4%), and severe stress (11.5%), people with high frequency of media use accounted for more.

The differences of residents' willingness to accept organ donation were statistically significant (*P* < 0.05) in terms of age, permanent residence, registered permanent residence, education level, marital status, number of houses, number of children, number of brothers and sisters, debt, religious belief, anxiety, depression and stress, etc., which indicated that these factors had significant influence on residents' willingness to accept organ donation.

### Scores of media use and willingness to accept organ donation

The scores of all scales of the included people were shown in Table 3: Among them, newspapers scored the lowest (1.86 ± 1.08) and smart phones scored the highest (4.33 ± 1.13). It showed that Chinese people were more inclined to smart phones in media use. The scores of the subjects' willingness to accept organ donation were moderate (56.93 ± 32.36), which indicated that Chinese residents' willingness to accept organ donation was average at present.

**Table 3:** The scores of media use and willingness to accept organ donation of the subjects.

**Scales**	**Items**	**Range of scores**	**M ±SD**
Newspaper	1	1–5	1.86 ± 1.08
Magazine	1	1–5	1.91 ± 1.05
Book (non-textbook)	1	1–5	2.73 ± 1.26
Broadcast	1	1–5	2.10 ± 1.19
TV	1	1–5	3.24 ± 1.28
PC (including tablet)	1	1–5	3.17 ± 1.44
Smartphone	1	1–5	4.33 ± 1.13
Organ donation acceptance willingness	1	0–100	56.93 ± 32.36

### Summary of residents' willingness to accept organ donation scores

**Figure 2:**
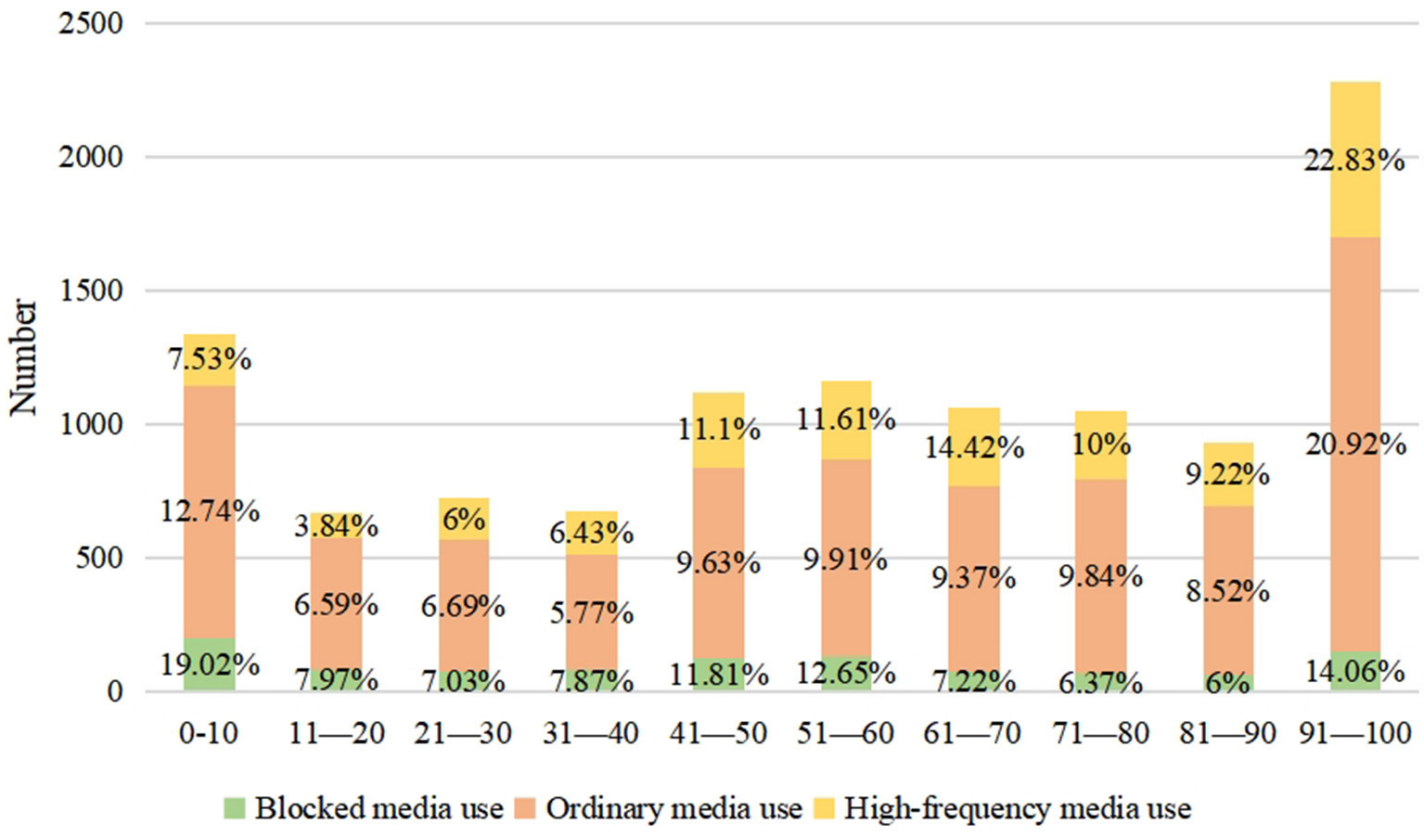
Summary of residents' willingness to donate organs.

In the summary of residents' willingness to accept organ donation scores (Figure 2), about 51.69% residents' willingness to accept organ donation scores were ≤ 60, and only about 20.70% residents' willingness to accept organ donation scores were between 91 and 100.

Among the organ donation intentions of the three categories of people who use media, people with “Ordinary media use” scores between 91 and 100 are the most (1,551), people with “High-frequency media use” followed (582), and people with “occluded media use” were the least (150). However, in their respective categories, “High-frequency media users” accounted for 22.83%, followed by “Ordinary media users” accounted for 20.92%, and “occluded media users” accounted for 14.06% of the lowest.

### Regression analysis of predictive variables on accept willingness of organ donation

As shown in Table 4, compared with ordinary media use, high-frequency media population (β = 0.06, *P* < 0.001) were positively correlated with their willingness to accept organ donation, residents who used media occlusion (β = −0.02, *P* < 0.001) were negatively correlated with their willingness to accept organ donation.

**Table 4:** Regression analysis of media use on the accept willingness to organ donation.

**Model**		**Unstandardized**		**Standardized**	***t***	***P***	**EXP(** ***β*** **) 95%**	
		**coefficients**		**coefficients**			**confidence interval**	
		**β**	**SE**	**β**			**LLCI**	**ULCI**
Independent variable	Media use (Ref: ordinary)	
	Occluded	−2.79	1.05	−0.02	−1.35	< 0.001	−3.81	−0.69
	High frequency	4.69	0.71	0.06	5.60	< 0.001	3.11	5.96
Control variable	Gender (Ref: Female)	
	Male	−2.38	0.60	−0.04	−4.00	< 0.001	−3.46	−1.11
	Age (Ref: ≤ 18)	
	≥66	−4.20	1.21	−0.04	−3.47	0.001	−10.44	−3.76
	Level of education (Ref: Primary school or below)	
	High school	5.96	1.13	0.09	5.26	< 0.001	3.23	7.78
	College level or above	9.93	1.16	0.15	8.59	< 0.001	7.52	12.31
	Household registration permit (Ref: Agriculture)	
	Non-agriculture	3.30	0.63	0.05	5.25	< 0.001	2.01	4.49
	Whether have children (Ref: No)	
	Yes	−7.40	0.65	−0.11	−11.34	< 0.001	−7.71	−4.32
	Whether have religious beliefs (Ref: No)	
	Yes	−5.12	1.76	−0.03	−2.91	0.004	−8.49	−1.59
	Pressure (Ref: Mild pressure)	
	Moderate pressure	3.91	0.71	0.06	5.54	< 0.001	2.58	5.34
	Severe pressure	21.01	1.37	0.15	15.29	< 0.001	18.50	23.90
	Social support	
	Family support	−0.62	0.11	−0.09	−5.47	< 0.001	−0.84	−0.39
	Friend support	0.95	0.12	0.14	8.19	< 0.001	0.69	1.15
	Other support	0.30	0.14	0.04	2.18	0.029	0.03	0.57

Residents with college level or above (β = 9.93, *P* < 0.001) and non-agricultural registered permanent residence (β = 3.30, *P* < 0.001) were more willing to accept organ donation. Male (β = −2.38, *P* < 0.001), older (β = −4.20, *P* = 0.001), residents with religious beliefs (β = −5.12, *P* = 0.004) were less willing to accept organ donation. Among individual social support, friend support (β = 0.95, *P* < 0.001) and other support (β = 0.30, *P* = 0.029) could enhance residents' willingness to accept organ donation. On the contrary, family support (β = −0.62, *P* < 0.001) could hinder residents' willingness to accept organ donation.

Among people with media use occlusion, residents with three or more houses (β = 0.08, *P* = 0.020), moderate anxiety (β = 0.07, *P* = 0.022), moderate stress (β = 0.15, *P* < 0.001), severe stress (β = 0.33, *P* < 0.001) and high support from friends (β = 0.26, *P* = 0.007) were more willing to accept organ donation. Other support (β = −0.23, *P* = 0.005) hindered residents' willingness to accept organ donation (see Table 5).

**Table 5:** Regression model of accept willingness to organ donation among people with media occlusion.

**Model**	**Unstandardized**		**Standardized**	***t***	***P***	**EXP(** ***β*** **) 95%**	
	**coefficients**		**coefficients**			**confidence interval**	
	**β**	**SE**	**β**			**LLCI**	**ULCI**
Number of houses owned (Ref:0)	
1	1.9	2.81	0.03	0.68	0.499	−3.57	7.46
2	6.35	3.63	0.08	1.75	0.081	−0.95	13.33
≥3	11.15	4.79	0.08	2.33	0.02	2.23	21.07
Anxiety (Ref: No anxiety)	
Mild anxiety	−0.37	2.17	−0.01	0.17	0.865	−5.92	5.33
Moderate anxiety	7.42	3.24	0.07	2.29	0.022	2.62	21.23
Severe anxiety	−0.79	6.81	−0.01	−0.12	0.907	−10.50	24.44
Pressure (Ref: Mild pressure)	
Moderate pressure	10.54	2.42	0.15	4.35	< 0.001	5.8	15.37
Severe pressure	34.91	3.87	0.33	9.03	< 0.001	27.63	42.85
Social support	
Family support	−0.15	0.41	0.03	−0.38	0.705	−0.98	0.62
Friend support	1.49	0.35	0.26	4.25	< 0.001	0.85	2.25
Other support	−1.27	0.45	−0.23	−2.83	0.005	−2.18	−0.42

Among people with ordinary media use, residents with college degree or above (β = 0.17, *P* < 0.001), non-agricultural registered permanent residence (β = 0.06, *P* < 0.001), mild anxiety (β = 0.04, *P* = 0.005), moderate anxiety (β = 0.05, *P* < 0.001) and severe stress (β = 0.07, *P* < 0.001) were more willing to accept organ donation. Male (β = −0.04, *P* < 0.001), had religious beliefs (β = −0.03, *P* = 0.002) and residents with children (β = −0.10, *P* < 0.001) were less willing to accept organ donation. In social support, friends' support (β = 0.12, *P* < 0.001) and other support (β = 0.07, *P* =0.002) increased residents' willingness to accept organ donation, family support (β = −0.10, *P* < 0.001) hindered residents' willingness to accept donations (see Table 6).

**Table 6:** Regression model of accept willingness to organ donation among ordinary media users.

**Model**	**Unstandardized**		**Standardized**	***t***	***P***	**EXP(** ***β*** **) 95%**	
	**coefficients**		**coefficients**			**confidence interval**	
	**β**	**SE**	**β**			**LLCI**	**ULCI**
Gender (Ref:Female)							
Male	−2.89	0.75	−0.04	−3.87	< 0.001	−4.36	−1.43
Age (Ref: ≤ 18)							
19–40	−3.43	1.37	−0.05	−2.51	0.012	−6.12	−0.75
41–65	−5.03	1.67	−0.07	−3.01	0.003	−8.31	−1.76
≥66	−8.68	2.56	−0.05	−3.39	0.001	−13.7	−3.66
Level of education (Ref: Primary school or below)							
High school	5.2	1.62	0.07	3.22	0.001	2.03	8.37
College level or above	11.29	1.68	0.17	6.71	< 0.001	7.99	14.58
Household registration permit (Ref:Agriculture)							
Non-agriculture	3.92	0.79	0.06	4.95	< 0.001	2.37	5.48
Whether have religious beliefs (Ref: No)							
Yes	−6.91	2.27	−0.03	−3.05	0.002	−11.4	−2.47
Whether have children (Ref: No)							
Yes	−6.84	1.09	−0.1	−6.25	< 0.001	−8.99	−4.7
Anxiety (Ref: No anxiety)							
Mild anxiety	2.45	0.86	0.04	2.86	0.004	0.76	4.13
Moderate anxiety	6.33	1.64	0.05	3.86	< 0.001	3.11	9.54
Severe anxiety	−1.21	3.08	−0.01	−0.39	0.694	−7.25	4.82
Pressure (Ref: Mild pressure)							
Moderate pressure	1.45	0.9	0.02	1.62	0.106	−0.31	3.22
Severe pressure	12.67	2.22	0.07	5.71	< 0.001	8.32	17.02
Social support							
Family support	−0.75	0.13	−0.1	5.61	< 0.001	−1.01	−0.49
Friend support	0.93	0.14	0.12	6.63	< 0.001	0.65	1.2
Other support	0.5	0.16	0.07	3.04	0.002	0.18	0.81

Among the high-frequency media users, the residents with college degree or above (β = 0.13, *P* = 0.001), moderate pressure (β = 0.09, *P* < 0.001) and severe pressure (β = 0.27, *P* < 0.001) were more willing to accept organ donation. Older residents (β = −0.11, *P* = 0.005) with high frequency of media exposure are less willing to accept organ donation (see Table 7).

**Table 7:** Regression model of accept willingness to organ donation among people high-frequency media user.

**Model**	**Unstandardized**		**Standardized**	***t***	***P***	**EXP(** ***β*** **) 95%**	
	**Coefficients**		**Coefficients**			**confidence interval**	
	**β**	**SE**	**β**			**LLCI**	**ULCI**
Age (Ref: ≤ 18)	
19–40	−3.27	2.34	−0.06	−1.4	0.162	−8.29	0.92
41–65	−4.17	2.35	−0.07	−1.78	0.076	−8.84	0.38
≥66	−10.9	2.82	−0.11	−3.85	< 0.001	−16.1	−4.99
Level of education (Ref: Primary school or below)	
High school	7.69	2.34	0.12	3.28	0.001	3.12	12.33
College level or above	7.9	2.28	0.13	3.46	0.001	3.91	12.89
Pressure (Ref: Mild pressure)	
Moderate pressure	5.83	1.46	0.09	4	< 0.001	1.93	7.62
Severe pressure	24.88	2.12	0.27	11.76	< 0.001	20.24	28.55

## Discussion

This study investigated and analyzed the influence of media use on Chinese residents' willingness to donate organs and other factors that may affect their willingness to accept organ donation. The study found that the degree of media use had a significant impact on residents' willingness to accept organ donation. “High-frequency media use” and “ordinary media use” had positive effects on organ donation willingness, “occluded media use” had a negative impact on organ donation willingness. In other words, the higher the degree of media use, the more willing to accept organ donation, and the lower the degree of media use, the lower the willingness to accept organ donation.

The content of media used might affect people's willingness to accept organ donation. Among the three categories of media use, the residents of “high-frequency media use” and “ordinary media use” were mainly young people, with the largest number of people using smart phones. There was an interactive platform of social media in smart phones, and social media played a certain role in increasing the effectiveness of living donation (33). For example, setting up online organ donation registration links (34), developing smart phone applications to increase living organ donation (35), implementing publicity and training programs to find living donor (36), etc. In addition, medical professionals in organ transplantation have begun to explore how to expand and educate the public through online platforms and social media (37). This made the smart phone-based “high-frequency media use” and “ordinary media use” people received more knowledge about organ donation, more objective understanding, and higher willingness to accept organ donation.

The residents with “media use occlusion” were mostly middle-aged and elderly people, and the media they were exposed to were mainly TV and radio. These media reports on organ donation issues were relatively lacking, which led to this group's little knowledge of organ donation and low willingness to accept organ donation. The findings of this study were inconsistent with a study conducted in Murcia, Spain. Television had the greatest influence on the public's awareness and attitude toward organ donation (38). Older people with lower education level were more likely to be affected by health problems depicted on TV (39). However, in China, TV, radio and other mass media seldom report on organ donation issues, and the low attention of Chinese mass media on organ donation issues had become the main restricting factor to improve the willingness to accept organ donation and the acceptance rate of organ transplantation (40).

The study also found some other factors that may affect residents' willingness to accept organ donation. Gender, age, whether have children or not, religious belief and so on all had negative influences on residents' willingness to accept organ donation. Education level, registered permanent residence nature, degree of stress, etc. all had positive influence on residents' willingness to accept organ donation. Studies showed that the younger (41) and better educated people (42) were more likely to make organ donation. Previous studies have found that men were less willing to donate and less likely to have conversations about organ donation, while women were more likely to mention their willingness to donate and their moral/altruistic/religious reasons (43). However, due to the lack of understanding of the religious aspects of organ donation and transplantation, many people rejected the concept of organ donation for religious reasons (44). The results were consistent with the data of this study.

Besides, social support was considered as a predictor of organ donation willingness (45). Social support is defined as information leading the subject to believe that he is cared for and loved, esteemed, and a member of a network of mutual obligations (46). The social support system aims to assess the views on the adequacy of social support from three specific sources: family, friends and other important sources (47). Individual attitude is not the only determinant of organ donation behavior (48). One of the major concerns about organ donation is the opposition of other people. Donors must deal with conflicts with their families in the decision-making process (49, 50), it will make the donors have ambivalence before organ donation. Social support can reduce the worries related to living donation and reduce the influence of worries on ambivalence (51), and then increase the willingness to accept organ donation. We found that the support of friends and other important people in social support had a significant positive impact on residents' willingness to accept organ donation, and the opinions or suggestions of friends and other important people could enhance people's willingness to accept organ donation. On the contrary, family support could hinder residents' willingness to accept organ donation to some extent. The reason was probably influenced by Chinese cultural environment. In the Chinese mind, everyone has a different distance from himself. The nearest others are family members, who usually have the strongest relationship with themselves, while the farthest others are unfamiliar members of society, who have the weakest relationship with themselves (52). The willingness to accept organ donation is often closely related to the closest family members. In “The Book of Filial Piety”, it was mentioned: “When the body is skinned, the parents are afraid to damage it, and filial piety begins.” Under the influence of Chinese traditional filial piety culture, family emotional factors will hinder people's willingness to accept organ donation to a certain extent.

In fact, the idea of “The Book of Filial Piety” was to oppose unnecessary damage to the body, but not to advocate absolute preservation of the body. Organ donation in modern society is aimed at helping others to treat patients and prolong their lives, and its loss to the body is positive, rather than meaningless (53). Since ancient times, China has emphasized “benevolence”. Altruistic organ donation is a virtue that emerged only today in the development of medical science, because it can save lives and give others a chance to be reborn (54). This is a typical “benevolence”. Many people only know that “the skin of the body is affected by the parents” is the absolute preservation of the body, but they don't know that it is a fearless injury against the body, which makes people not willing to accept organ donation. Therefore, the media can design some publicity contents aiming at Chinese traditional culture when conducting popular science propaganda on organ donation, so as to alleviate or dispel citizens' concerns about the traditional concept of organ donation, with a view to increasing the organ donation rate.

### Research highlights and limitations

This study is the first survey of residents' willingness to accept organ donation in Chinese mainland, with a large sample size and wide representation. In addition, this study takes media use as an independent variable for the first time and classifies people, so as to explore the degree of influence of different residents' media use on their willingness to accept organ donation, which is innovative.

This study also has some limitations: firstly, this study uses cross-sectional data as the data source, so it is difficult to make causal inference. Secondly, due to the limitation of sampling methods, there may be sample selection bias.

## Conclusion

It is suggested that the government and relevant departments should pay more attention to the willingness of people with different media usage levels to accept organ donation, formulate personalized and targeted dissemination strategies for organ donation health information for different media usage groups, and focus on different public groups with different media usage characteristics.

## Data availability statement

The original contributions presented in the study are included in the article/supplementary files, further inquiries can be directed to the corresponding authors.

## Ethics statement

The studies involving human participants were reviewed and approved by the Institutional Review Committee of Jinan University (JNUKY-2021-018), Guangdong, China. The participants provided their written informed consent to participate in this study.

## Author contributions

FG and YJ provided the formal analysis, writing—original draft, and writing—editing. YW and XS provided the conceptualization and methodology. FG and YW provided the validation. JZ, MC, XJ, and YW provided the writing—review. All authors contributed to the article and approved the submitted version.

## Funding

The scientific research project of Shaanxi Provincial Education Department in 2021-the key research base project of philosophy and social sciences (Grant No. 21JZ017). It is the achievement of the National Cultural Research Center along the Southwest Silk Road of the State Ethnic Affairs Commission.

## Conflict of interest

The authors declare that the research was conducted in the absence of any commercial or financial relationships that could be construed as a potential conflict of interest.

## Publisher's note

All claims expressed in this article are solely those of the authors and do not necessarily represent those of their affiliated organizations, or those of the publisher, the editors and the reviewers. Any product that may be evaluated in this article, or claim that may be made by its manufacturer, is not guaranteed or endorsed by the publisher.
